# Addressing vulnerability of pregnant refugees

**DOI:** 10.2471/BLT.17.193664

**Published:** 2017-09-01

**Authors:** Mary Malebranche, Kara Nerenberg, Amy Metcalfe, Gabriel E Fabreau

**Affiliations:** a Department of Medicine, University of Calgary, Calgary, Alberta T2N 4N1, Canada.; b Department of Obstetrics & Gynecology, University of Calgary, Calgary, Canada.

The number of refugees – defined as individuals fleeing war, violence or a well-founded fear of persecution based on race, religion, nationality, political opinion or membership in a particular social group^1^ – is currently estimated at an unprecedented 22.5 million.^2^ Countries resettling refugees urgently need to address the specific mental, physical and reproductive health needs of the growing number of refugee women. This is particularly important during pregnancy, when the vulnerability of refugee women is notably heightened, increasing their risk for largely preventable adverse maternal and newborn outcomes. In September 2016, during the United Nations Summit for Refugees and Migrants held in New York, the importance of tackling the specific vulnerabilities of refugee women was recognized by the United Nations General Assembly through the adoption of the New York Declaration for Refugees and Migrants,^3^ marking an international call to action.

As Canadian health-care practitioners, our shared perspective is on the vital role high-income countries such as Canada can play in optimizing the health of refugee women. Although the majority of global refugees are currently hosted by low- and middle-income countries,^4^ high-income countries with established refugee resettlement programmes such as Canada, Germany and Sweden have dramatically increased their resettlement efforts. This is reflected in a 77% increase in the number of refugees who resettled in high-income countries between 2015 and 2016.^4^ However, studies that have explored health outcomes among resettled refugee women in countries such as Australia, Canada and Sweden demonstrate significant disparities in maternal and perinatal outcomes when compared to non-displaced women.^5^^–^^7^ Disparities include higher rates of preterm birth, low birth-weight infants, stillbirths and maternal mortality.^5^^–^^7^ These adverse outcomes do not only impact mothers and their newborns in the immediate perinatal period: growing evidence suggests that adverse birth outcomes can have long-lasting repercussions on the health and development of the newborn well into adulthood. For example, infants born preterm are at increased risk for developing chronic diseases such as hypertension, diabetes and cardiovascular disease later in life.^8^ Moreover, evidence suggests that not only are first-generation migrant populations at increased risk of developing mental illness, but that this risk may persist into the second generation of resettled families.^9^ Therefore, failing to address the complex health and social needs of pregnant refugee women resettling in high-income countries means missing valuable opportunities to optimize health across two generations.

By implementing and ensuring accessibility to evidence-based health-care interventions, we can work to mitigate adverse maternal and newborn outcomes for refugee women resettling in high-income countries. Many of these adverse outcomes are related to largely preventable risk factors such as suboptimal health prior to conception, short inter-pregnancy intervals and difficulty accessing timely prenatal care, thus presenting opportunities to identify and treat preexisting maternal conditions. However, due to multiple factors including language barriers, cultural differences and difficulty navigating complex health and social systems in host countries, refugee women tend to have delayed initiation of prenatal care and fewer prenatal visits.^10^ By addressing these barriers and ensuring refugee women have access to culturally sensitive reproductive care from conception to the postpartum period, we can reduce modifiable risk factors and strive to eliminate disparities in maternal and newborn outcomes.

Moreover, interventions that improve maternal and newborn outcomes are cost–effective in the long term.^11^ The direct and indirect costs incurred across the lifespan associated with high-risk pregnancies, preterm deliveries and low birth-weight infants are markedly higher than those associated with healthy term infants.^12^ With developed nations increasingly challenged by ever-expanding health care expenditures, investing in targeted and cost–effective interventions, such as prenatal care for vulnerable populations – namely refugee women – is vital. Failing to do so may result in long-lasting morbidity for refugee mothers and their newborns and lead to costly long-term sequelae.

In the context of the current global refugee crisis and international calls to protect the rights of refugees and eliminate striking disparities in the maternal and perinatal outcomes of refugee women, we must take urgent action to address their unmet health needs, particularly during the highly vulnerable time surrounding pregnancy. Through targeted, evidence-based and cost–effective interventions, we can ensure refugee women and their children are ultimately afforded equal opportunity to realize their full educational, social and economic potential as they rebuild their lives in their new homes. Though we have focused specifically on the role of high-income countries in doing so, this issue is global. Across high-, middle- and low-income countries alike, we cannot afford to ignore the United Nations’ call to action to protect and care for refugee women.
